# Highlights from the Respiratory Failure and Mechanical Ventilation 2020 Conference

**DOI:** 10.1183/23120541.00752-2020

**Published:** 2021-02-08

**Authors:** Adelaide Withers, Tiffany Choi Ching Man, Rebecca D'Cruz, Heder de Vries, Christoph Fisser, Carla Ribeiro, Neeraj Shah, Marine Van Hollebecke, Bettine A.H. Vosse, Leo Heunks, Maxime Patout

**Affiliations:** 1 Respiratory Medicine, Perth Children's Hospital, Perth, Australia; 2 School of Health Sciences, Caritas Institute of Higher Education, Tseung Kwan O, New Territories, Hong Kong; 3 Lane Fox Clinical Respiratory Physiology Centre, Guy's and St Thomas’ NHS Foundation Trust, London, UK; 4 Centre for Human and Applied Physiological Sciences (CHAPS), King's College London, London, UK; 5 Intensive Care Department, Amsterdam UMC, location VUmc, Amsterdam, The Netherlands; 6 Dept of Internal Medicine II, University Hospital Regensburg, Regensburg, Germany; 7 Pulmonology Dept, Centro Hospitalar de Vila Nova de Gaia/Espinho, Vila Nova de Gaia, Portugal; 8 Dept of Rehabilitation Sciences, KU Leuven, Leuven, Belgium; 9 Dept of Pulmonology, Maastricht University Medical Centre, Maastricht, The Netherlands; 10 Centre of Home Mechanical Ventilation Maastricht, Maastricht University Medical Centre, Maastricht, The Netherlands; 11 AP-HP, Groupe Hospitalier Universitaire APHP-Sorbonne Université, site Pitié-Salpêtrière, Service des Pathologies du Sommeil (Département R3S), Paris, France; 12 Sorbonne Université, INSERM, UMRS1158 Neurophysiologie Respiratoire Expérimentale et Clinique, Paris, France

## Abstract

The Respiratory Intensive Care Assembly of the European Respiratory Society organised the first Respiratory Failure and Mechanical Ventilation Conference in Berlin in February 2020. The conference covered acute and chronic respiratory failure in both adults and children. During this 3-day conference, patient selection, diagnostic strategies and treatment options were discussed by international experts. Lectures delivered during the event have been summarised by Early Career Members of the Assembly and take-home messages highlighted.

## Abstract 2

**During #RFMV2020, patient selection, diagnostic strategies and treatment options were discussed by international experts. This review summarises the most important take-home messages.** https://bit.ly/3murkoa

## Introduction

A detailed understanding of respiratory physiology is the foundation of adequate patient management, as highlighted in the opening session by Martin Tobin, Leo Heunks and Stefano Nava.

Martin Tobin first gave a historical perspective on respiratory medicine. He presented how the principles of mechanical ventilation were described in the 16th century; however, they are reportedly alluded to in the Old Testament of the Bible. Mechanical positive pressure ventilation further developed in the 18th and 19th centuries with bellow ventilation, but was thought to be deleterious because of pneumothoraxes. Negative-pressure ventilation was invented in the second half of the 19th century and was the main method of delivering mechanical ventilation during the poliomyelitis epidemic in Copenhagen. Prof. Tobin remarked that intensive care medicine began during this pandemic thanks to Bjørn Ibsen, an anaesthesiologist who recognised that polio was causing respiratory acidosis and treated patients with positive-pressure ventilation delivered through tracheostomy [1]. This lecture was particularly enlightening given the imminent declaration of the severe acute respiratory syndrome coronavirus 2/coronavirus disease 2019 pandemic.

Leo Heunks provided an overview of the neural respiratory drive and its three feedback mechanisms: chemical, cortical and reflex, both in healthy subjects and pathological states. As neural drive can preclude lung-protective ventilation, he described how it can be evaluated. Furthermore, he described how neural drive is influenced by propofol sedation, opioids, extracorporeal carbon dioxide (CO_2_) removal and adjustments in ventilator settings. The importance of understanding respiratory mechanics was highlighted, as this has direct consequences for lung-protective ventilation strategies. In addition to plateau pressure, the importance of the driving pressure on the onset of ventilator-induced lung injury was highlighted.

Stefano Nava closed the session with an overview of the mechanisms of hypoxaemia including 1) alveolo–capillary diffusion limitation, 2) ventilation/perfusion mismatch, 3) hypoventilation, 4) shunt and 5) reduced inspired oxygen tension. He showed how these mechanisms are implicated in respiratory diseases. He stressed that the understanding of these mechanisms was important to deliver adequate ventilatory support to patients.

**Take-home message**

Understanding the physiology of respiratory disease and the principles of ventilation is key to delivering optimal clinical care.

## Acute respiratory failure

### High-flow nasal therapy and noninvasive ventilation

#### Hypoxaemic acute respiratory failure

High-flow nasal therapy (HFNT) is widely applied to treat hypoxaemic acute respiratory failure in clinical practice, despite currently limited data on patient selection, settings and monitoring. HFNT may improve oxygenation, reduce respiratory rate and work of breathing compared to conventional oxygen [2]. However, its effect on intubation risk appears to be dependent on the aetiology and severity of acute respiratory failure [3–5]. The ROX (respiratory rate and oxygenation) index (respiratory rate to peripheral oxygen saturation/inspiratory oxygen fraction (*F*_IO_2__) ratio) may be used to predict HFNT failure and avoid intubation delays [6].

#### Noninvasive ventilation in acute hypercapnic respiratory failure

Noninvasive ventilation (NIV) is established in the management of acute hypercapnic respiratory failure [7] and reduces mortality, admission duration and healthcare costs compared to invasive ventilation [8]. It can be delivered on general wards, high-dependency and intensive care units [9–11]. However, staff experience predicts success [12] as does adequate patient selection. Early recognition of treatment failure is crucial to avoid delayed intubation [13]. Helmets, which require higher pressure support [14], are increasingly applied [11], with no observed differences in treatment success compared to oronasal masks [15].

#### Noninvasive ventilation in acute hypercapnic respiratory failure in non-COPD patients

Prospective randomised trials on NIV in obesity-related acute hypercapnic respiratory failure are lacking. Morbid obesity, pneumonia and multiorgan failure may predict treatment failure [16], and endotracheal intubation must be readily available [17]. Post-discharge domiciliary NIV or continuous positive airway pressure may reduce short-term mortality [18]. NIV may also be effective in acute hypercapnic respiratory failure in a range of neuromuscular diseases [19, 20] and as a bridge to lung transplantation in cystic fibrosis [21].

#### HFNRT in acute hypercapnic respiratory failure

HFNT prolongs expiratory time and decreases respiratory rate and work of breathing in stable hypercapnic patients [22]. Trials evaluating its effects in acute hypercapnic respiratory failure compared to NIV [23] and during acute hypercapnic respiratory failure breaks compared to conventional oxygen are ongoing [24]. To date, there are insufficient data to support its routine application in clinical practice. Data on HFNT physiological and clinical effects in acute hypercapnic respiratory failure, patient selection and settings are needed to guide clinical practice.

#### NIV use post-extubation

Extubation failure increases mortality and duration of hospitalisation [25]. Systematic application of post-extubation NIV may increase mortality, probably due to delayed reintubation [26, 27]. However, among high-risk patients (older age, hypercapnic during spontaneous breathing trial, chronic lung disease, surgical), NIV can prevent post-extubation acute hypercapnic respiratory failure and reintubation [28–31], particularly when administered with HFNT [32].

#### NIV in palliative care

NIV may be offered to palliate breathlessness in life-limiting disease [7, 33, 34]. It may reduce opiate requirements [35] without impairing quality of life [36, 37]. However, risks of distress from alarms, prolonged hospitalisations for training and impaired communication and oral intake must be anticipated. Alternative therapies, including mechanical insufflation–exsufflation, HFNT and Tai Chi may be considered [38]. Planned NIV withdrawal to alleviate distress may be performed with analgaesia/sedation and oxygen at home or in hospital [39].

**Take-home messages**

- HFNT may be used to improve oxygenation in hypoxaemic acute respiratory failure.
- Following careful patient selection, NIV is an effective therapy for hypercapnic respiratory failure and improves a range of clinical outcomes.
- Patients receiving HFNT and NIV must be monitored closely by experienced staff for signs of treatment failure.

### Adverse effects of mechanical ventilation

Mechanical ventilation is key for the management of severe acute respiratory failure, but can also have adverse effects that need to be monitored.

#### Heart–lung interaction

Positive-pressure ventilation has both beneficial and adverse effects on the cardiovascular system. Positive-pressure ventilation changes lung volumes, and more importantly, increases intrathoracic pressure. The intrathoracic pressure has an important influence on venous return, a major determinant of cardiac output. Positive-pressure ventilation decreases pre-load, increases right ventricular afterload (increased pulmonary vascular resistance) and decreases left ventricular afterload. Hence, mechanical ventilation can induce right ventricle dysfunction. Physicians should also take into account right ventricle function in order to set positive end-expiratory pressure (PEEP) according to right ventricle function [40].

#### Ventilator-induced lung injury

Lung injury in patients on mechanical ventilation is closely related to ventilator settings. Indeed, ventilation set at low lung volumes can cause atelectrauma, whereas ventilation set at high lung volumes can lead to overdistension and barotrauma [41]. There is convincing evidence for benefit of ventilation with a low tidal volume in patients with acute respiratory distress syndrome (ARDS). Such benefit may also be seen in patients without ARDS [42–44]. Female patients with ARDS tend to have higher mortality due to higher tidal volume (per predicted bodyweight) compared to males [45, 46].

#### Ventilator-induced respiratory muscle injury

Mechanical ventilation can injure the respiratory muscles as well as the lungs. Diaphragm dysfunction occurs very frequently in the intensive care unit (ICU) and results from different mechanisms related to the consecutive time points in the disease course of the ventilated patient. It is associated with weaning failure and decreased survival [47]. Ultrasound of the diaphragm may be helpful in monitoring diaphragm function and effort in the ICU [48].

#### Long-term consequences of mechanical ventilation

Critical illness leads to multimorbidity ranging from functional impairment caused by muscle weakness to neurocognitive dysfunction and mood disorders associated with diminished quality of life [49–51]. Critical illness affects patients and their families. Caregivers are at high risk of mood disorders and increased mortality [52]. Attempts should be made to identify at-risk patients and caregivers and long-term risk assessments should be part of standard care. An integrated post-ICU care pathway across the care and recovery continuum should be a new practice standard to meet complex patient and caregiver needs after critical illness [53].

**Take-home messages**

- Ventilator-induced lung injury can be limited by careful selection of lung-protective ventilation.
- Diaphragm-induced injury develops frequently and may be limited by monitoring effort, for instance with diaphragm ultrasound.
- Critical care survivors and their caregivers may suffer from long-term sequelae that should be monitored.

### Rescue therapies for respiratory failure

#### Lung recruitment manoeuvres or prone positioning

Lung recruitment manoeuvres are typically performed by temporarily increasing airway pressure.

A recent trial performing a lung recruitment manoeuvre and subsequent PEEP titration in ARDS resulted in increased mortality [54]. Therefore, recruitment manoeuvres should only be used as a rescue therapy and not as a routine manoeuvre in patients with hypoxaemic failure. The use of higher PEEP compared to lower PEEP failed to show a benefit on mortality [55–57].

Prone positioning is another method that aims to enhance lung recruitment, and as such homogenise the lung in patients with ARDS. A more homogenous lung is less likely to be affected by shear stress and atelectrauma during tidal breathing. The study by Gattinoni *et al.* [58] on prone positioning in ARDS showed no survival benefit. However, a randomised controlled study with more prolonged prone positioning (>16 h·day^−1^) showed improved survival [59, 60].

#### Extracorporeal CO_2_ removal

From a physiological perspective, arterial carbon dioxide tension (*P*_aCO_2__) reduction leads to respiratory muscle unloading. Extracorporeal CO_2_ removal may be used in COPD patients to avoid intubation as rescue therapy after NIV failure or to facilitate weaning. However, robust data supporting its use in daily clinical practice are currently lacking.

#### Extracorporeal membrane oxygenation

Two large randomised controlled trials have evaluated extracorporeal membrane oxygenation (ECMO) in patients with severe ARDS. The CESAR trial showed an improved survival compared in patients referred to ECMO centres compared to treatment in non-ECMO centres [61]. The EOLIA trial did not show any improvement [62]. However, in this trial, the rate of crossover to ECMO was 28%. *Post hoc* analyses reported significant relative risk reduction with ECMO [63].

**Take-home messages**

- Prone positioning is effective in improving survival in patients with moderate-to-severe ARDS (arterial oxygen tension/*F*_IO_2__ ratio <150 mmHg).
- The use of extracorporeal CO_2_ removal needs to be supported by randomised controlled trials.
- ECMO may improve survival in patients with severe ARDS.

### Diagnostic techniques

#### Lung ultrasound

Lung ultrasound is an appealing bedside diagnostic tool, since it is a safe, easy-to-learn, fast, accurate, repeatable and real-time technique that avoids ionising radiation exposure and transfer-related risks. Lung ultrasound requires a simple machine and any probe may be used. Both eight- and 12-zone scanning approaches are generally adopted.

There is evidence for usefulness and accuracy of lung ultrasound in diagnosing interstitial syndrome, consolidation, pneumothorax, pleural effusion and acute respiratory failure. In addition, lung ultrasound is suitable for monitoring aeration changes and effect of treatment in the critically ill. However, it has limitations in detecting small lesions and in patients with subcutaneous emphysema/oedema, obesity, large dressings, bandages and drains.

#### Oesophageal pressure monitoring

The measurements of oesophageal pressure (*P*_oes_) is minimally invasive in mechanically ventilated patients. *P*_oes_ estimates transpulmonary pressure and the intensity of patient breathing effort [64].

*P*_oes_ measurements enhance our understanding of the pathophysiology of ARDS, patient–ventilator interaction and weaning failure [64]. There is emerging evidence that supports the use of *P*_oes_ in patients with ARDS [65].

#### Bronchoscopy in mechanically ventilated patients

Bronchoscopy in invasively ventilated patients allows inspection of the airway, sampling and treatment (such as foreign body removal). However, it can also have detrimental consequences (table 1). Given the frailty of patients with ARDS and given the possible complications, bronchoscopy should only be performed following care consideration of risks and benefits. Expected benefits will be assessed using the diagnosis hypothesis, considering alternative noninvasive diagnostic tests, the immunological status of the patient and risks of infection.

**Take-home messages**

- Lung ultrasound is an easy, safe way to assess lung parenchyma and pleural space at the bedside.
- *P*_oes_ measurements help to understand pathophysiological changes and aid decision making to improve ventilatory support.
- Benefits and risks must be assessed before performing bronchoscopy in invasively ventilated patients.

**TABLE 1:** Physiological consequences of bronchoscopy

**Respiratory consequences**	**Circulatory consequences**	**Other consequences**
Increased airway resistance	Decreased cardiac output	Increased intracranial pressure
Decreased lung compliance	Increased heart rate	
Impaired gas exchange	Increased pulmonary arterial pressure	

### Year in preview in acute respiratory failure

Marcus Schultz identified five paradigm shifts that will influence research in acute respiratory failure in the coming years.

The first paradigm shift is to move from “mad physiology” to evidence-based logical thinking. For years we have tried to “restore” clinical parameters of critically ill patients to match healthy subjects; for example, the arterial blood gas parameters of ARDS patients can be brought to near-normal levels by administering high tidal volumes, but mortality goes up drastically [66]. Instead, perhaps we should rest the lung to minimise mortality [67].

The second important paradigm shift is to incorporate personalised medicine in our trials and treatments. Trials on PEEP-setting might be beneficial if we can differentiate recruitable from non-recruitable patients, as opposed to giving large groups of heterogeneous patients the same level of PEEP [68]. Patients with ARDS might have different disease phenotypes (focal *versus* non-focal) that might require different treatment [69]. Computed tomography scans, lung ultrasound or labs-on-a-chip are exciting techniques to differentiate ARDS phenotypes. The same principle might even apply to sepsis.

The third paradigm shift is to stop thinking about the lung as a sterile environment, and to further study the importance of the lung microbiome. Several studies have observed associations between the lung microbiome and ICU outcomes [70]. Conversely, several interventions that optimise the lung microbiome have shown promising results [71, 72].

The fourth paradigm shift is to evaluate whether we treat females and males equally well. Recent retrospective analyses have found that females tend to receive higher tidal volumes, which might have detrimental effects on outcome [73].

Lastly, physicians should be prepared to have smart algorithms assist them with various clinical tasks, such as managing the settings of the mechanical ventilator. This will allow the clinician to focus on other tasks.

**Take-home messages**

- Stop “mad physiology”: critically ill patients do not need to be normalised.
- Differentiation of disease phenotypes can lead to personalisation of ICU treatment.
- The lung microbiome provides an exciting new target to prevent infections in ventilated patients.
- Sex differences in research and clinical care must be minimised.
- There is an emerging role for artificial intelligence in clinical practice and research.

## Chronic respiratory failure

### NIV in COPD patients

#### Patient selection

The use of home NIV to treat COPD patients was only recently supported by large-scale randomised controlled trials. A meta-analysis confirmed its efficacy in decreasing all-cause mortality and hospital admissions. However, health-related quality of life did not significantly improve [74]. Based on results from randomised controlled trials, home NIV should be used [75] in 1) stable severe COPD patients who have persistent hypercapnia in stable state [76] and 2) patients with severe COPD who were admitted for acute hypercapnic respiratory failure and who remained hypercapnic 2–4 weeks following discharge [77] (table 2). In these two trials, ventilation was set in order to significantly reduce daytime *P*_aCO_2__ (−0.5 kPa or −20%).

**Take-home messages**

- Home NIV reduces mortality in stable COPD patients with daytime *P*_aCO_2__ >52 mmHg/6.9 kPa.
- Home NIV increases time to readmission in COPD or death patients with daytime *P*_aCO_2__ >53 mmHg/7 kPa by 2–4 weeks following an episode of acute hypercapnic respiratory failure.

**TABLE 2:** Overview of selection criteria for noninvasive ventilation (NIV) in patients with COPD

	**Who?**	**When?**	**How?**	**Why?**
**Stable COPD**	Severe stable COPD (FEV_1_ <1 L) Baseline *P*_aCO_2__ >52 mmHg/6.9 kPa Preserved exercise capacity (6MWT >200 m) Low annual emergency admission rate	Stable state Low annual emergency admission rate	Targeted *P*_aCO_2__ reduction	Reduces 1-year all-cause mortality
**Post-AECOPD**	Severe COPD (FEV_1_ <1 L) Following a life-threatening exacerbation of COPD requiring acute NIV Persistent hypercapnic respiratory failure defined by *P*_aCO_2__ >53 mmHg/7 kPa 2–4 weeks post-AECOPD	2–4 weeks post-AECOPD if *P*_aCO_2__ >52 mmHg	Targeted *P*_aCO_2__ reduction	Increases admission-free survival Cost-effective treatment

FEV_1_: forced expiratory volume in 1 s; *P*_aCO_2__: arterial carbon dioxide tension; 6MWT: 6-min walk test; AECOPD: acute exacerbation of COPD.

#### What are the targets?

NIV is set to reduce *P*_aCO_2__. However, improvement in health-related quality of life or of sleep and in ability to perform daily life activities are more important for patients. Patient-centred goals such as sleep quality, sputum clearance, health-related quality of life, exercise capacity, survival and exacerbation frequency are improved with NIV [76–78]. Reduction of exacerbation and hospital admission are also meaningful targets, as they have a detrimental effect on disease course. Close monitoring of NIV tolerance should be conducted, as ventilation side-effects are frequent and may influence efficacy and adherence to the treatment. Good adherence to ventilation (>4 h·night^−1^) is associated with better survival [79].

**Take-home messages**

- NIV must aim to improve meaningful patient-centred outcomes in addition to reducing *P*_aCO_2__.
- Compliance to NIV needs to be monitored, as well as NIV-related side-effects.

#### ERS task force on long-term NIV in COPD [75]

In addition to defining patients in whom NIV should be initiated, the ERS task force suggested using high-intensity NIV with high inspiratory airway pressures (IPAP) instead of low IPAP, which is ineffective in reducing *P*_aCO_2__ and might even reduce health-related quality of life [80, 81].

### NIV in patients with neuromuscular disease

#### Role of neuromuscular diseases in pulmonary medicine

As highlighted in the opening session, neuromuscular diseases (NMDs) such as poliomyelitis [1] are an important part of mechanical ventilation. Rose *et al.* [82] demonstrated the continued importance of NMDs, with an increasing prevalence and decreasing mortality in a national Canadian survey. Of all patients with a NMD, a third receive pulmonary input, with frequent comorbid airway disease [83].

The early studies by Simonds and co-workers demonstrated that survival was significantly longer in NMD patients compared with COPD [84] and that NIV categorically prolongs survival in the presence of hypercapnia in Duchenne muscular dystrophy [85]. Importantly, Kohler *et al.* [86] demonstrated that the introduction of NIV did not have a detrimental effect on health-related quality of life in patients with NMD.

**Take-home messages**

- NMDs are a common indication for initiation of NIV.
- NIV improves survival and quality of life in patients with NMD.

#### NIV in rapidly progressive NMD: when to start?

**FIGURE 1:**
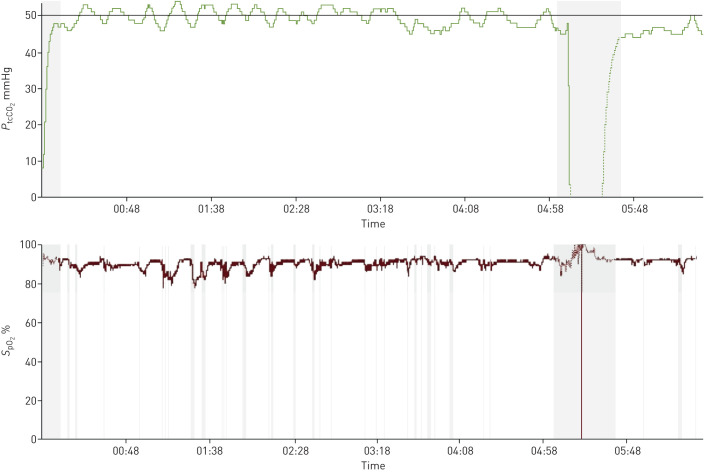
Overnight transcutaneous capnography during self-ventilation of a patient with type I myotonic dystrophy showing severe hypoventilation without rapid oxygen desaturation that would suggest obstructive sleep apnoea. *P*_tcCO_2__: transcutaneous carbon dioxide tension; *S*_pO_2__: peripheral oxygen saturation.

A prognostic model for death in amyotrophic lateral sclerosis was highlighted as a method to predict time of initiation [87]. Before initiation of ventilation polygraphy and/or capnography (figure 1) should be used to screen for hypoventilation, with polysomnography being reserved for unusual cases. An algorithm for setting up NIV in amyotrophic lateral sclerosis by Morélot-Panzini *et al.* [88] illustrated when and how NIV should be set up in patients with amyotrophic lateral sclerosis. In amyotrophic lateral sclerosis, there is noninferiority of daytime NIV set-up compared with conventional polysomnography set-up [89].

**Take-home messages**

- Overnight assessment is useful to adequately initiate NIV in patients with NMDs.
- NIV can be initiated as an outpatient in patients with NMDs.

#### Cough assistance

In patients with NMD, cough is frequently impaired and secretion clearance techniques are required [90]. The use of mechanical insufflation–exsufflation is effective at increasing peak cough flow [91], but fatigue is significantly increased immediately after therapy [92]. The physiological effectiveness of mechanical insufflation–exsufflation remains to be elucidated with more work needed on its effect on breathing pattern and lung mechanics [93, 94].

**Take-home message**

- Mechanical insufflation–exsufflation improves secretion clearance.

### A year in review in chronic respiratory failure

Wolfram Windisch analysed three questions that will shape the future of chronic respiratory failure and long-term home ventilation [75].

The first is whether outpatient control of long-term noninvasive mechanical ventilation is feasible in COPD patients. Recent studies suggest that two-thirds of patients can be managed without hospitalisation [95].

The next question is whether NIV at home is beneficial after an exacerbation in COPD patients. Although this strategy improves arterial blood gases, studies have conflicting results regarding readmission rates, mortality and long-term quality of life [77, 96].

The final question was whether newer modes of mechanical ventilation are beneficial in the long-term home ventilation setting. Although there is a strong physiological rationale for newer modes, evidence so far has been conflicting.

**Take-home messages**

- Outpatient management of NIV in COPD patients is feasible, cost-effective and preferred by patients.
- More studies are required to decide the role of home ventilation after episodes of acute respiratory failure in COPD patients; better patient selection will increase the benefit of high-intensity home ventilation.
- Further study is warranted to increase the quality of life of patients receiving home ventilation.

## Paediatric mechanical ventilation

### Particularities

Managing respiratory failure and ventilation in paediatrics presents numerous and unique challenges due to heterogenous causes of respiratory failure, significant variation in age, size and respiratory mechanics of children, lack of appropriate equipment, ethical issues of long-term invasive ventilation when children cannot contribute to decision making and difficulties assessing quality of life.

These factors make extrapolating findings from adult clinical trials problematic; additionally, conducting large randomised control trials in children is challenging, leading to a lack of evidence to guide management. These complexities were highlighted, emphasising the lack of a “one guideline fits all” approach with illustrative cases. Sessions focused on lung-protective ventilation strategies in children, novel methods of optimising ventilation and use of long-term invasive and noninvasive ventilation in the home.

### Lung-protective ventilation

Lung-protective ventilation strategies can be instituted from birth. Evidence-based recommendations for premature neonates include using gestation to guide choice of oxygen or room air during resuscitation and targeting normal post-natal saturations of preterm infants. Avoiding invasive mechanical ventilation is preferred where possible, with early use of continuous positive airway pressure and rescue surfactant administered by less-invasive surfactant therapy or minimally invasive surfactant treatment techniques that do not require intubation. Recommended mechanical ventilation techniques are volume-targeted/volume guarantee modes to limit volutrauma, particularly after rapid changes in compliance post-surfactant administration.

There may be an increased capacity of the paediatric lung for repair, possibly reducing vulnerability to ventilator-induced lung injury. Recommendations from the Pediatric Acute Lung Injury Consensus Conference included use of low tidal volumes adjusted for pulmonary compliance, maintaining inspiratory plateau pressure <28 cmH_2_O and permissive hypercapnia for moderate-to-severe paediatric ARDS. Heterogenous causes of paediatric ARDS makes determining optimal PEEP difficult. Interestingly, Khemani *et al*. [97] showed that paediatricians often used lower PEEP than recommended by ARDSNet and this was associated with higher mortality. The role of NIV appears to be limited to mild paediatric ARDS.

The only paediatric randomised control trial of prone positioning was stopped early due to futility.

**Take-home messages**

- In premature neonates, invasive ventilation should be avoided if possible.
- Protective ventilation in paediatric ARDS differs from adult ARDS.

### New modes of ventilation in acute respiratory failure

High-frequency oscillatory ventilation in paediatric ARDS has been shown to be associated with adverse outcomes, especially increased risk of air leak. Hopefully, the PROSPECT trial (Prone and Oscillation Paediatric Clinical Trial) will clarify the roles of high-frequency oscillatory ventilation and prone positioning in the management of paediatric ARDS.

Reducing the mechanical power applied to the lung during mechanical ventilation with ECMO and paying particular attention to very low tidal volumes, low respiratory rates and low plateau pressures may reduce lung injury, with different strategies for obstructive lung disease and early and late ARDS.

Neurally adjusted ventilation assist can be particularly useful in complex children with severe tracheomalacia, neuromuscular weakness, post-cardiac surgery and ECMO. It can be used to improve patient–ventilator synchrony with NIV.

HFNT is increasingly being used to treat acute respiratory failure in paediatrics, with the majority of evidence in infants with bronchiolitis. There is some evidence that it may be useful in other forms of acute respiratory failure, but has yet to be shown to be superior to NIV. Increasingly, HFNT is being used in the home for children unable to tolerate continuous positive airway pressure for obstructive sleep apnoea, particularly children with trisomy 21 and craniofacial disorders.

**Take-home messages**

- The use of neurally adjusted ventilation and high-frequency oscillatory ventilation is not supported by controlled trials.
- HFNT is widely used for the management of bronchiolitis.
- HFNT can be an alternative to continuous positive airway pressure.

### The year in preview in paediatric mechanical ventilation

Brigitte Fauroux pointed out that many treatment recommendations and protocols in paediatric medicine are not based on evidence, but on expert opinion [98]. Several factors contribute to this problem. Many of the current recommendations in paediatric mechanical ventilation are based on studies conducted in the adult population [98]. However, the physiology of the paediatric population might differ substantially from adults. Paediatric researchers should focus on trials on lung-protective ventilation, assisted spontaneous breathing, NIV and weaning [99].

Recent trials in these areas have been promising, but require more validation in larger cohorts and in more countries and centres. For instance, a small single-centre study observed benefit for permissive hypercapnia [100]. The CALIPSO trial found that survival is not better when surfactant is administered to paediatric patients with lung injury [101]. Another study observed a staggering two-fold increase in mortality when using airway pressure-release ventilation *versus* conventional low tidal volume ventilation in paediatric patients [102]. Prone positioning (a trusted method in adults) was studied in paediatrics, and was found to increase lung homogeneity in one-third of the participants [103].

Lastly, the importance of sleep in the paediatric ICU is under-studied. This is troublesome, as lower sleep quality has been linked with susceptibility to infection and higher rates of failing spontaneous breathing trials [104]. Simple interventions such as better sound and light management might make a lot of difference for sleep quality and should be studied. Additionally, cerebral oxygenation during respiratory events might be of paramount importance for neurological outcomes and requires further study [105], possibly by using new techniques such as near-infrared spectroscopy [106].

**Take-home messages**

- Validation of opinion-based recommendations is paramount for evidence-based paediatric medicine.
- Trials in paediatrics should switch from “hard end-points”, such as mortality, to more relevant outcome parameters, such as neurological development.
- The importance of sleep quality and cerebral oxygenation need to be studied further in paediatric medicine.

## References

[1] Ibsen B The anaesthetist's viewpoint on the treatment of respiratory complications in poliomyelitis during the epidemic in Copenhagen, 1952. Proc R Soc Med 1954; 47: 72–74. 13134176 10.1177/003591575404700120 PMC1918820

[2] Mauri T, Turrini C, Eronia N, et al. Physiologic effects of high-flow nasal cannula in acute hypoxemic respiratory failure. Am J Respir Crit Care Med 2017; 195: 1207–1215. doi:10.1164/rccm.201605-0916OC 27997805

[3] Frat J-P, Thille AW, Mercat A, et al. High-flow oxygen through nasal cannula in acute hypoxemic respiratory failure. N Engl J Med 2015; 372: 2185–2196. doi:10.1056/NEJMoa1503326 25981908

[4] Azoulay E, Lemiale V, Mokart D, et al. Effect of high-flow nasal oxygen vs standard oxygen on 28-day mortality in immunocompromised patients with acute respiratory failure: the HIGH randomized clinical trial. JAMA 2018; 320: 2099–2107. doi:10.1001/jama.2018.14282 30357270 PMC6583581

[5] Rochwerg B, Granton D, Wang DX, et al. High flow nasal cannula compared with conventional oxygen therapy for acute hypoxemic respiratory failure: a systematic review and meta-analysis. Intensive Care Med 2019; 45: 563–572. doi:10.1007/s00134-019-05658-2 30888444

[6] Roca O, Caralt B, Messika J, et al. An index combining respiratory rate and oxygenation to predict outcome of nasal high-flow therapy. Am J Respir Crit Care Med 2019; 199: 1368–1376. doi:10.1164/rccm.201803-0589OC 30576221

[7] Rochwerg B, Brochard L, Elliott MW, et al. Official ERS/ATS clinical practice guidelines: noninvasive ventilation for acute respiratory failure. Eur Respir J 2017; 50: 1602426. doi:10.1183/13993003.02426-2016 28860265

[8] Lindenauer PK, Stefan MS, Shieh M-S, et al. Outcomes associated with invasive and noninvasive ventilation among patients hospitalized with exacerbations of chronic obstructive pulmonary disease. JAMA Intern Med 2014; 174: 1982–1993. doi:10.1001/jamainternmed.2014.5430 25347545 PMC4501470

[9] Fiorino S, Bacchi-Reggiani L, Detotto E, et al. Efficacy of non-invasive mechanical ventilation in the general ward in patients with chronic obstructive pulmonary disease admitted for hypercapnic acute respiratory failure and pH < 7.35: a feasibility pilot study. Intern Med J 2015; 45: 527–537. doi:10.1111/imj.12726 25684643

[10] Plant PK, Owen JL, Elliott MW Early use of non-invasive ventilation for acute exacerbations of chronic obstructive pulmonary disease on general respiratory wards: a multicentre randomised controlled trial. Lancet 2000; 355: 1931–1935. doi:10.1016/S0140-6736(00)02323-0 10859037

[11] Crimi C, Noto A, Princi P, et al. A European survey of noninvasive ventilation practices. Eur Respir J 2010; 36: 362–369. doi:10.1183/09031936.00123509 20075052

[12] Contou D, Fragnoli C, Córdoba-Izquierdo A, et al. Noninvasive ventilation for acute hypercapnic respiratory failure: intubation rate in an experienced unit. Respir Care 2013; 58: 2045–2052. doi:10.4187/respcare.02456 23737546

[13] Ozyilmaz E, Ozsancak Ugurlu A, Nava S Timing of noninvasive ventilation failure: causes, risk factors, and potential remedies. BMC Pulm Med 2014; 14: 19. doi:10.1186/1471-2466-14-19 24520952 PMC3925956

[14] Vargas F, Thille A, Lyazidi A, et al. Helmet with specific settings *versus* facemask for noninvasive ventilation. Crit Care Med 2009; 37: 1921–1928. doi:10.1097/CCM.0b013e31819fff93 19384209

[15] Pisani L, Mega C, Vaschetto R, et al. Oronasal mask *versus* helmet in acute hypercapnic respiratory failure. Eur Respir J 2015; 45: 691–699. doi:10.1183/09031936.00053814 25504992

[16] Lemyze M, Taufour P, Duhamel A, et al. Determinants of noninvasive ventilation success or failure in morbidly obese patients in acute respiratory failure. PLoS One 2014; 9: e97563. doi:10.1371/journal.pone.0097563 24819141 PMC4018299

[17] Duarte AG, Justino E, Bigler T, et al. Outcomes of morbidly obese patients requiring mechanical ventilation for acute respiratory failure. Crit Care Med 2007; 35: 732–737. doi:10.1097/01.CCM.0000256842.39767.41 17255878

[18] Mokhlesi B, Masa JF, Afshar M, et al. The effect of hospital discharge with empiric noninvasive ventilation on mortality in hospitalized patients with obesity hypoventilation syndrome. An individual patient data meta-analysis. Ann Am Thorac Soc 2020; 17: 627–637. doi:10.1513/AnnalsATS.201912-887OC 32023419

[19] Racca F, Del Sorbo L, Mongini T, et al. Respiratory management of acute respiratory failure in neuromuscular diseases. Minerva Anestesiol 2010; 76: 51–62. 20125073

[20] Flandreau G, Bourdin G, Leray V, et al. Management and long-term outcome of patients with chronic neuromuscular disease admitted to the intensive care unit for acute respiratory failure: a single-center retrospective study. Respir Care 2011; 56: 953–960. doi:10.4187/respcare.00862 21740726

[21] Madden BP, Kariyawasam H, Siddiqi AJ, et al. Noninvasive ventilation in cystic fibrosis patients with acute or chronic respiratory failure. Eur Respir J 2002; 19: 310–313. doi:10.1183/09031936.02.00218502 11866011

[22] Pisani L, Fasano L, Corcione N, et al. Change in pulmonary mechanics and the effect on breathing pattern of high flow oxygen therapy in stable hypercapnic COPD. Thorax 2017; 72: 373–375. doi:10.1136/thoraxjnl-2016-209673 28104830

[23] Cortegiani A, Longhini F, Carlucci A, et al. High-flow nasal therapy *versus* noninvasive ventilation in COPD patients with mild-to-moderate hypercapnic acute respiratory failure: study protocol for a noninferiority randomized clinical trial. Trials 2019; 20: 450. doi:10.1186/s13063-019-3514-1 31331372 PMC6647141

[24] Ricard J-D, Dib F, Esposito-Farese M, et al. Comparison of high flow nasal cannula oxygen and conventional oxygen therapy on ventilatory support duration during acute-on-chronic respiratory failure: study protocol of a multicentre, randomised, controlled trial. The “HIGH-FLOW ACRF” study. BMJ Open 2018; 8: e022983. 10.1136/bmjopen-2018-022983 PMC6150142 30232113

[25] Thille AW, Richard JC, Brochard L The decision to extubate in the intensive care unit. Am J Respir Crit Care Med 2013; 187: 1294–1302. doi:10.1164/rccm.201208-1523CI 23641924

[26] Esteban A, Frutos-Vivar F, Ferguson ND, et al. Noninvasive positive-pressure ventilation for respiratory failure after extubation. N Engl J Med 2004; 350: 2452–2460. doi:10.1056/NEJMoa032736 15190137

[27] Su CL, Chiang LL, Yang SH, et al. Preventive use of noninvasive ventilation after extubation: a prospective, multicenter randomized controlled trial. Respir Care 2012; 57: 204–210. 21762554 10.4187/respcare.01141

[28] Ferrer M, Valencia M, Nicolas JM, et al. Early noninvasive ventilation averts extubation failure in patients at risk: a randomized trial. Am J Respir Crit Care Med 2006; 173: 164–170. doi:10.1164/rccm.200505-718OC 16224108

[29] Nava S, Gregoretti C, Fanfulla F, et al. Noninvasive ventilation to prevent respiratory failure after extubation in high-risk patients. Crit Care Med 2005; 33: 2465–2470. doi:10.1097/01.CCM.0000186416.44752.72 16276167

[30] Thille AW, Harrois A, Schortgen F, et al. Outcomes of extubation failure in medical intensive care unit patients. Crit Care Med 2011; 39: 2612–2618. doi:10.1097/CCM.0b013e3182282a5a 21765357

[31] Jaber S, Lescot T, Futier E, et al. Effect of noninvasive ventilation on tracheal reintubation among patients with hypoxemic respiratory failure following abdominal surgery: a randomized clinical trial. JAMA 2016; 315: 1345–1353. doi:10.1001/jama.2016.2706 26975890

[32] Thille AW, Muller G, Gacouin A, et al. Effect of postextubation high-flow nasal oxygen with noninvasive ventilation *vs* high-flow nasal oxygen alone on reintubation among patients at high risk of extubation failure: a randomized clinical trial. JAMA 2019; 322: 1465–1475. doi:10.1001/jama.2019.14901 31577036 PMC6802261

[33] Curtis JR, Cook DJ, Sinuff T, et al. Noninvasive positive pressure ventilation in critical and palliative care settings: understanding the goals of therapy. Crit Care Med 2007; 35: 932–939. doi:10.1097/01.CCM.0000256725.73993.74 17255876

[34] Hui D, Morgado M, Chisholm G, et al. High-flow oxygen and bilevel positive airway pressure for persistent dyspnea in patients with advanced cancer: a phase II randomized trial. J Pain Symptom Manage 2013; 46: 463–473. doi:10.1016/j.jpainsymman.2012.10.284 23739633 PMC3795985

[35] Nava S, Ferrer M, Esquinas A, et al. Palliative use of non-invasive ventilation in end-of-life patients with solid tumours: a randomised feasibility trial. Lancet Oncol 2013; 14: 219–227. doi:10.1016/S1470-2045(13)70009-3 23406914

[36] Vilaça M, Aragão I, Cardoso T, et al. The role of noninvasive ventilation in patients with “do not intubate” order in the emergency setting. PLoS One 2016; 11: e0149649. doi:10.1371/journal.pone.0149649 26901060 PMC4763309

[37] Bourke SC, Tomlinson M, Williams TL, et al. Effects of non-invasive ventilation on survival and quality of life in patients with amyotrophic lateral sclerosis: a randomised controlled trial. Lancet Neurol 2006; 5: 140–147. doi:10.1016/S1474-4422(05)70326-4 16426990

[38] Hui EST, Cheng JOY, Cheng HKT Benefits of Tai Chi in palliative care for advanced cancer patients. Palliat Med 2008; 22: 93–94. doi:10.1177/0269216307084610 18216084

[39] Faull C, Rowe Haynes C, Oliver D Issues for palliative medicine doctors surrounding the withdrawal of non-invasive ventilation at the request of a patient with motor neurone disease: a scoping study. BMJ Support Palliat Care 2014; 4: 43–49. doi:10.1136/bmjspcare-2013-000470 24644770

[40] Repessé X, Charron C, Vieillard-Baron A Acute cor pulmonale in ARDS: rationale for protecting the right ventricle. Chest 2015; 147: 259–265. doi:10.1378/chest.14-0877 25560864

[41] Slutsky AS, Ranieri VM Ventilator-induced lung injury. N Engl J Med 2013; 369: 2126–2136. doi:10.1056/NEJMra1208707 24283226

[42] Bellani G, Laffey JG, Pham T, et al. Epidemiology, patterns of care, and mortality for patients with acute respiratory distress syndrome in intensive care units in 50 countries. JAMA 2016; 315: 788–800. doi:10.1001/jama.2016.0291 26903337

[43] Neto AS, Barbas CSV, Simonis FD, et al. Epidemiological characteristics, practice of ventilation, and clinical outcome in patients at risk of acute respiratory distress syndrome in intensive care units from 16 countries (PRoVENT): an international, multicentre, prospective study. Lancet Respir Med 2016; 4: 882–893. doi:10.1016/S2213-2600(16)30305-8 27717861

[44] Pisani L, Algera AG, Serpa Neto A, et al. PRactice of VENTilation in Middle-Income Countries (PRoVENT-iMIC): rationale and protocol for a prospective international multicentre observational study in intensive care units in Asia. BMJ Open 2018; 8: e020841. doi:10.1136/bmjopen-2017-020841 PMC5931304 29705765

[45] LAS VEGAS investigators Epidemiology, practice of ventilation and outcome for patients at increased risk of postoperative pulmonary complications: LAS VEGAS – an observational study in 29 countries. Eur J Anaesthesiol 2017; 34: 492–507. doi:10.1097/EJA.0000000000000646 28633157 PMC5502122

[46] McNicholas BA, Madotto F, Pham T, et al. Demographics, management and outcome of females and males with acute respiratory distress syndrome in the LUNG SAFE prospective cohort study. Eur Respir J 2019; 54: 1900609. doi:10.1183/13993003.00609-2019 31346004

[47] Demoule A, Jung B, Prodanovic H, et al. Diaphragm dysfunction on admission to the intensive care unit. Prevalence, risk factors, and prognostic impact – a prospective study. Am J Respir Crit Care Med 2013; 188: 213–219. doi:10.1164/rccm.201209-1668OC 23641946

[48] Dres M, Demoule A Monitoring diaphragm function in the ICU. Curr Opin Crit Care 2020; 26: 18–25. doi:10.1097/MCC.0000000000000682 31876624

[49] Herridge MS, Cheung AM, Tansey CM, et al. One-year outcomes in survivors of the acute respiratory distress syndrome. N Engl J Med 2003; 348: 683–693. doi:10.1056/NEJMoa022450 12594312

[50] Hopkins RO, Herridge MS Quality of life, emotional abnormalities, and cognitive dysfunction in survivors of acute lung injury/acute respiratory distress syndrome. Clin Chest Med 2006; 27: 679–689. doi:10.1016/j.ccm.2006.06.003 17085255

[51] Pandharipande PP, Girard TD, Jackson JC, et al. Long-term cognitive impairment after critical illness. N Engl J Med 2013; 369: 1306–1316. doi:10.1056/NEJMoa1301372 24088092 PMC3922401

[52] Cameron JI, Chu LM, Matte A, et al. One-year outcomes in caregivers of critically ill patients. N Engl J Med 2016; 374: 1831–1841. doi:10.1056/NEJMoa1511160 27168433

[53] Herridge MS, Chu LM, Matte A, et al. The RECOVER program: disability risk groups and 1-year outcome after 7 or more days of mechanical ventilation. Am J Respir Crit Care Med 2016; 194: 831–844. doi:10.1164/rccm.201512-2343OC 26974173

[54] Writing Group for the Alveolar Recruitment for Acute Respiratory Distress Syndrome Trial (ART) Investigators, Cavalcanti AB, Suzumura ÉA, et al. Effect of lung recruitment and titrated positive end-expiratory pressure (PEEP) *vs* low PEEP on mortality in patients with acute respiratory distress syndrome: a randomized clinical trial. JAMA 2017; 318: 1335–1345. doi:10.1001/jama.2017.14171 28973363 PMC5710484

[55] Mercat A, Richard J-CM, Vielle B, et al. Positive end-expiratory pressure setting in adults with acute lung injury and acute respiratory distress syndrome: a randomized controlled trial. JAMA 2008; 299: 646–655. doi:10.1001/jama.299.6.646 18270353

[56] Brower RG, Lanken PN, MacIntyre N, et al. Higher *versus* lower positive end-expiratory pressures in patients with the acute respiratory distress syndrome. N Engl J Med 2004; 351: 327–336. doi:10.1056/NEJMoa032193 15269312

[57] Meade MO, Cook DJ, Guyatt GH, et al. Ventilation strategy using low tidal volumes, recruitment maneuvers, and high positive end-expiratory pressure for acute lung injury and acute respiratory distress syndrome: a randomized controlled trial. JAMA 2008; 299: 637–645. doi:10.1001/jama.299.6.637 18270352

[58] Gattinoni L, Tognoni G, Pesenti A, et al. Effect of prone positioning on the survival of patients with acute respiratory failure. N Engl J Med 2001; 345: 568–573. doi:10.1056/NEJMoa010043 11529210

[59] Guérin C, Reignier J, Richard J-C, et al. Prone positioning in severe acute respiratory distress syndrome. N Engl J Med 2013; 368: 2159–2168. doi:10.1056/NEJMoa1214103 23688302

[60] Gattinoni L, Taccone P, Carlesso E, et al. Prone position in acute respiratory distress syndrome. Rationale, indications, and limits. Am J Respir Crit Care Med 2013; 188: 1286–1293. doi:10.1164/rccm.201308-1532CI 24134414

[61] Peek GJ, Mugford M, Tiruvoipati R, et al. Efficacy and economic assessment of conventional ventilatory support *versus* extracorporeal membrane oxygenation for severe adult respiratory failure (CESAR): a multicentre randomised controlled trial. Lancet 2009; 374: 1351–1363. doi:10.1016/S0140-6736(09)61069-2 19762075

[62] Combes A, Hajage D, Capellier G, et al. Extracorporeal membrane oxygenation for severe acute respiratory distress syndrome. N Engl J Med 2018; 378: 1965–1975. doi:10.1056/NEJMoa1800385 29791822

[63] Gattinoni L, Vasques F, Quintel M Use of ECMO in ARDS: does the EOLIA trial really help? Crit Care 2018; 22: 171. doi:10.1186/s13054-018-2098-6 29976250 PMC6034241

[64] Akoumianaki E, Maggiore SM, Valenza F, et al. The application of esophageal pressure measurement in patients with respiratory failure. Am J Respir Crit Care Med 2014; 189: 520–531. doi:10.1164/rccm.201312-2193CI 24467647

[65] Talmor D, Sarge T, Malhotra A, et al. Mechanical ventilation guided by esophageal pressure in acute lung injury. N Engl J Med 2008; 359: 2095–2104. doi:10.1056/NEJMoa0708638 19001507 PMC3969885

[66] Acute Respiratory Distress Syndrome Network, Brower RG, Matthay MA, et al. Ventilation with lower tidal volumes as compared with traditional tidal volumes for acute lung injury and the acute respiratory distress syndrome. N Engl J Med 2000; 342: 1301–1308. doi:10.1056/NEJM200005043421801 10793162

[67] Pelosi P, Rocco PRM, Gama de Abreu M Close down the lungs and keep them resting to minimize ventilator-induced lung injury. Crit Care 2018; 22: 72. doi:10.1186/s13054-018-1991-3 29558993 PMC5861643

[68] Gattinoni L, Caironi P, Cressoni M, et al. Lung recruitment in patients with the acute respiratory distress syndrome. N Engl J Med 2006; 354: 1775–1786. doi:10.1056/NEJMoa052052 16641394

[69] Constantin J-M, Jabaudon M, Lefrant J-Y, et al. Personalised mechanical ventilation tailored to lung morphology *versus* low positive end-expiratory pressure for patients with acute respiratory distress syndrome in France (the LIVE study): a multicentre, single-blind, randomised controlled trial. Lancet Respir Med 2019; 7: 870–880. doi:10.1016/S2213-2600(19)30138-9 31399381

[70] Dickson RP The microbiome and critical illness. Lancet Respir Med 2016; 4: 59–72. doi:10.1016/S2213-2600(15)00427-0 26700442 PMC4752077

[71] Manzanares W, Lemieux M, Langlois PL, et al. Probiotic and synbiotic therapy in critical illness: a systematic review and meta-analysis. Crit Care 2016; 19: 262. doi:10.1186/s13054-016-1434-y 27538711 PMC4991010

[72] Johnstone J, Heels-Ansdell D, Thabane L, et al. Evaluating probiotics for the prevention of ventilator-associated pneumonia: a randomised placebo-controlled multicentre trial protocol and statistical analysis plan for PROSPECT. BMJ Open 2019; 9: e025228. doi:10.1136/bmjopen-2018-025228 PMC6596980 31227528

[73] Schultz MJ, Karagiannidis C Is gender inequity in ventilator management a “women's issue”? Eur Respir J 2019; 54: 1901588. doi:10.1183/13993003.01588-2019 31624130

[74] Wilson ME, Dobler CC, Morrow AS, et al. Association of home noninvasive positive pressure ventilation with clinical outcomes in chronic obstructive pulmonary disease: a systematic review and meta-analysis. JAMA 2020; 323: 455–465. doi:10.1001/jama.2019.22343 32016309 PMC7042860

[75] Ergan B, Oczkowski S, Rochwerg B, et al. European Respiratory Society guidelines on long-term home non-invasive ventilation for management of COPD. Eur Respir J 2019; 54: 1901003. doi:10.1183/13993003.01003-2019 31467119

[76] Köhnlein T, Windisch W, Köhler D, et al. Non-invasive positive pressure ventilation for the treatment of severe stable chronic obstructive pulmonary disease: a prospective, multicentre, randomised, controlled clinical trial. Lancet Respir Med 2014; 2: 698–705. doi:10.1016/S2213-2600(14)70153-5 25066329

[77] Murphy PB, Rehal S, Arbane G, et al. Effect of home noninvasive ventilation with oxygen therapy *vs* oxygen therapy alone on hospital readmission or death after an acute COPD exacerbation: a randomized clinical trial. JAMA 2017; 317: 2177–2186. doi:10.1001/jama.2017.4451 28528348 PMC5710342

[78] Duiverman ML, Wempe JB, Bladder G, et al. Nocturnal non-invasive ventilation in addition to rehabilitation in hypercapnic patients with COPD. Thorax 2008; 63: 1052–1057. doi:10.1136/thx.2008.099044 18710905

[79] Patout M, Lhuillier E, Kaltsakas G, et al. Long-term survival following initiation of home non-invasive ventilation: a European study. Thorax 2020; 75: 965–973. doi:10.1136/thoraxjnl-2019-214204 32895315

[80] Struik FM, Lacasse Y, Goldstein RS, et al. Nocturnal noninvasive positive pressure ventilation in stable COPD: a systematic review and individual patient data meta-analysis. Respir Med 2014; 108: 329–337. doi:10.1016/j.rmed.2013.10.007 24157199

[81] Dreher M, Storre JH, Schmoor C, et al. High-intensity *versus* low-intensity non-invasive ventilation in patients with stable hypercapnic COPD: a randomised crossover trial. Thorax 2010; 65: 303–308. doi:10.1136/thx.2009.124263 20388753

[82] Rose L, McKim D, Leasa D, et al. Trends in incidence, prevalence, and mortality of neuromuscular disease in Ontario, Canada: a population-based retrospective cohort study (2003–2014). PLoS One 2019; 14: e0210574. 30913206 10.1371/journal.pone.0210574 PMC6435115

[83] Rose L, McKim D, Leasa D, et al. Patterns of healthcare utilisation for respiratory complications of adults with neuromuscular disease: a population study. Eur Respir J 2018; 52: 1800754. doi:10.1183/13993003.00754-2018 30139772

[84] Simonds AK, Elliott MW Outcome of domiciliary nasal intermittent positive pressure ventilation in restrictive and obstructive disorders. Thorax 1995; 50: 604–609. doi:10.1136/thx.50.6.604 7638799 PMC1021256

[85] Simonds AK, Muntoni F, Heather S, et al. Impact of nasal ventilation on survival in hypercapnic Duchenne muscular dystrophy. Thorax 1998; 53: 949–952. doi:10.1136/thx.53.11.949 10193393 PMC1745110

[86] Kohler M, Clarenbach CF, Böni L, et al. Quality of life, physical disability, and respiratory impairment in Duchenne muscular dystrophy. Am J Respir Crit Care Med 2005; 172: 1032–1036. doi:10.1164/rccm.200503-322OC 15961695

[87] Ackrivo J, Hansen-Flaschen J, Wileyto EP, et al. Development of a prognostic model of respiratory insufficiency or death in amyotrophic lateral sclerosis. Eur Respir J 2019; 53: 1802237. doi:10.1183/13993003.02237-2018 30728207 PMC6684229

[88] Morélot-Panzini C, Bruneteau G, Gonzalez-Bermejo J NIV in amyotrophic lateral sclerosis: the “when” and “how” of the matter. Respirology 2019; 24: 521–530. doi:10.1111/resp.13525 30912216

[89] Hannan LM, Rautela L, Berlowitz DJ, et al. Randomised controlled trial of polysomnographic titration of noninvasive ventilation. Eur Respir J 2019; 53: 1802118. doi:10.1183/13993003.02118-2018 30880286

[90] Chatwin M, Toussaint M, Gonçalves MR, et al. Airway clearance techniques in neuromuscular disorders: a state of the art review. Respir Med 2018; 136: 98–110. doi:10.1016/j.rmed.2018.01.012 29501255

[91] Winck JC, Gonçalves MR, Lourenço C, et al. Effects of mechanical insufflation–exsufflation on respiratory parameters for patients with chronic airway secretion encumbrance. Chest 2004; 126: 774–780. doi:10.1378/chest.126.3.774 15364756

[92] Chatwin M, Simonds AK The addition of mechanical insufflation/exsufflation shortens airway-clearance sessions in neuromuscular patients with chest infection. Respir Care 2009; 54: 1473–1479. 19863831

[93] Morrow B, Zampoli M, van Aswegen H, et al. Mechanical insufflation–exsufflation for people with neuromuscular disorders. Cochrane Database Syst Rev 2013; 12: CD010044. 10.1002/14651858.CD010044.pub2 24374746

[94] Toussaint M, Gonçalves M, Chatwin M Effects of mechanical insufflation–exsufflation on the breathing pattern in stable subjects with Duchenne muscular dystrophy: a step in a wrong direction. Respir Care 2019; 64: 235–236. doi:10.4187/respcare.06495 30705147

[95] Schwarz SB, Callegari J, Hamm C, et al. Is outpatient control of long-term non-invasive ventilation feasible in chronic obstructive pulmonary disease patients? Respiration 2018; 95: 154–160. doi:10.1159/000484569 29232680

[96] Struik FM, Sprooten RTM, Kerstjens HAM, et al. Nocturnal non-invasive ventilation in COPD patients with prolonged hypercapnia after ventilatory support for acute respiratory failure: a randomised, controlled, parallel-group study. Thorax 2014; 69: 826–834. doi:10.1136/thoraxjnl-2014-205126 24781217

[97] Khemani RG, Parvathaneni K, Yehya N, et al. Positive end-expiratory pressure lower than the ARDS Network protocol is associated with higher pediatric acute respiratory distress syndrome mortality. Am J Respir Crit Care Med 2018; 198: 77–89. doi:10.1164/rccm.201707-1404OC 29373802 PMC6034123

[98] Kneyber MCJ, de Luca D, Calderini E, et al. Recommendations for mechanical ventilation of critically ill children from the Paediatric Mechanical Ventilation Consensus Conference (PEMVECC). Intensive Care Med 2017; 43: 1764–1780. doi:10.1007/s00134-017-4920-z 28936698 PMC5717127

[99] Rimensberger PC, Cheifetz IM, Kneyber MCJ The top ten unknowns in paediatric mechanical ventilation. Intensive Care Med 2018; 44: 366–370. doi:10.1007/s00134-017-4847-4 28555411

[100] Fuchs H, Rossmann N, Schmid MB, et al. Permissive hypercapnia for severe acute respiratory distress syndrome in immunocompromised children: a single center experience. PLoS One 2017; 12: e0179974. 28632754 10.1371/journal.pone.0179974 PMC5478142

[101] Thomas NJ, Spear D, Wasserman E, et al. CALIPSO: a randomized controlled trial of calfactant for acute lung injury in pediatric stem cell and oncology patients. Biol Blood Marrow Transplant 2018; 24: 2479–2486. doi:10.1016/j.bbmt.2018.07.023 30059785 PMC10479933

[102] Lalgudi Ganesan S, Jayashree M, Chandra Singhi S, et al. Airway pressure release ventilation in pediatric acute respiratory distress syndrome. A randomized controlled trial. Am J Respir Crit Care Med 2018; 198: 1199–1207. doi:10.1164/rccm.201705-0989OC 29641221

[103] Lupton-Smith A, Argent A, Rimensberger P, et al. Prone positioning improves ventilation homogeneity in children with acute respiratory distress syndrome. Pediatr Crit Care Med 2017; 18: e229–e234. doi:10.1097/PCC.0000000000001145 28328787

[104] Dres M, Younes M, Rittayamai N, et al. Sleep and Pathological Wakefulness at the Time of Liberation from Mechanical Ventilation (SLEEWE). A prospective multicenter physiological study. Am J Respir Crit Care Med 2019; 199: 1106–1115. doi:10.1164/rccm.201811-2119OC 30818966

[105] Tabone L, Khirani S, Olmo Arroyo J, et al. Cerebral oxygenation during respiratory events in children with sleep-disordered breathing and associated disorders. J Pediatr 2019; 214: 134–140. doi:10.1016/j.jpeds.2019.07.040 31540763

[106] Wood MD, Jacobson JA, Maslove DM, et al. The physiological determinants of near-infrared spectroscopy-derived regional cerebral oxygenation in critically ill adults. Intensive Care Med Exp 2019; 7: 23. doi:10.1186/s40635-019-0247-0 31049754 PMC6497723

